# Faculty development programs improve the quality of Multiple Choice Questions items' writing

**DOI:** 10.1038/srep09556

**Published:** 2015-04-01

**Authors:** Hamza Mohammad Abdulghani, Farah Ahmad, Mohammad Irshad, Mahmoud Salah Khalil, Ghadeer Khalid Al-Shaikh, Sadiqa Syed, Abdulmajeed Abdurrahman Aldrees, Norah Alrowais, Shafiul Haque

**Affiliations:** 1 Department of Medical Education, King Saud University, Riyadh-11321, Saudi Arabia; 2 Department of Obstetrics & Gynecology, King Saud University, Riyadh-11321, Saudi Arabia; 3 Department of Basic Sciences, The Princess Nourah bint Abdulrahman University, Riyadh, Saudi Arabia; 4 Department of Family & Community Medicine, King Saud University, Riyadh-11321, Saudi Arabia; 5 Research and Scientific Studies Unit, College of Nursing and Allied Health Sciences, Jazan University, Jazan-45142, Saudi Arabia

## Abstract

The aim of this study was to assess the utility of long term faculty development programs (FDPs) in order to improve the quality of multiple choice questions (MCQs) items' writing. This was a quasi-experimental study, conducted with newly joined faculty members. The MCQ items were analyzed for difficulty index, discriminating index, reliability, Bloom's cognitive levels, item writing flaws (IWFs) and MCQs' nonfunctioning distractors (NFDs) based test courses of respiratory, cardiovascular and renal blocks. Significant improvement was found in the difficulty index values of pre- to post-training (p = 0.003). MCQs with moderate difficulty and higher discrimination were found to be more in the post-training tests in all three courses. Easy questions were decreased from 36.7 to 22.5%. Significant improvement was also reported in the discriminating indices from 92.1 to 95.4% after training (p = 0.132). More number of higher cognitive level of Bloom's taxonomy was reported in the post-training test items (p<0.0001). Also, NFDs and IWFs were reported less in the post-training items (p<0.02). The MCQs written by the faculties without participating in FDPs are usually of low quality. This study suggests that newly joined faculties need active participation in FDPs as these programs are supportive in improving the quality of MCQs' items writing.

Faculty development is defined as the process designed to prepare and enhance the productivity of academic staff for other pertinent roles, such as teaching, assessment, research, managerial and administrative issues^1^ as well as the development of resource material and facilitation which are required for active and student-centered learning^2^. Students' learning is largely driven and enhanced by assessment, thus development of high quality test is an important skill for educators^3^. The mode of assessment has been shown to influence the students' learning capabilities^4^. Usually, educators develop the test items by themselves or sometimes rely on item test banks as a source of questions. The possibility of error is more in case of test banks if their staff members are not well educated and professionally trained enough for the development of test items^5^. Hence, the assessment tool should be valid and reliable, and capable of measuring the diverse characteristics of professional competencies. Multiple choice questions (MCQs) are one of the most frequently used competency test type. MCQs are appropriate competency test for measuring knowledge, comprehension and can be designed to measure application and analysis^6^. Use of well-designed MCQs has been increased significantly due to their higher reliability, validity, and ease of scoring^7^,^8^. Also, well-constructed MCQs are capable of testing the higher levels of cognitive reasoning and can efficiently discriminate between high- and low-achieving students^9^,^10^. Despite the above said facts pertaining to well-constructed MCQ items, various studies have documented violation of MCQs' construction guidelines^9^,^11^.

Generally, faculty members may be asked to perform duties for which they have received no formal training and experience^12^. Faculty development programs (FDPs) are therefore required to provide wide range of learning opportunities available to academic staff ranging from conferences on education to informal discussions on the development of assessment materials to support the running courses. However, a successful FDP requires more than simple attendance; a degree of reflection and development is also needed to ensure continuity for personal development and the desired outcomes which improve the teaching, learning and assessment process^13^.

FDPs can be evaluated by the Kirkpatrick's model^14^. The Kirkpatrick's model describes four levels of outcome, i.e., learners' reaction (to the educational experience); learning (which refers to acquisition of new knowledge and skills); behavior (which refers to changes in practice and the application of learning to practice); and results (which refers to change at the level of the learner and the organization as the main outcome of a program)^15^. However, only scanty studies have reported the fourth level of Kirkpatrick's model^16^,^17^,^18^.

Teachers' training in the 21^st^ century needs to be widen its spotlight by using varied learning methods based on established learning theories, fostering partnerships and collaboration, and thoroughly evaluating interventions to keep pace with the changes in medical curricula^19^. In order to address the above mentioned needs, the Faculty Development Unit, Department of Medical Education, College of Medicine, King Saud University (KSU), Saudi Arabia conducted two workshop training programs in the academic year 2013–2014 for all newly joined faculty members (demonstrators, lecturers, assistant professors, associate professors and professors) who were involved in teaching of various subjects of medicine for the first year in the College of Medicine, Princess Nourah University (PNU), Riyadh, Saudi Arabia. The main focus of the workshops' training program was to construct appropriate MCQ items by the participants based upon the sound scientific standard and guidelines. The long term impacts of FDPs have been evaluated with pre-program, immediate post-program and follow-up study^14^,^20^. Therefore, the present study aims to evaluate the effect of long term well-structured FDPs in order to improve the quality of MCQs items' writing.

## Results

The results of the final MCQs based examinations of all the three courses (respiratory, cardiovascular, and renal) for academic years 2012–2013 (before workshop training) and 2013–2014 (after workshop training) were analyzed separately. The reliability co-efficient (Kr-20) of the all three examinations before and after workshop training program were more than 0.92. The improvement of students average mean score of the three courses before workshop training (mean score 18.29 ± 0.77) and after workshop training (mean score 20.33 ± 0.47) was observed (Table 1). Similarly, overall passing rate of students increased from 49.2 to 56.3% after workshop training.

The difficulty index (P) and discrimination index (DI) values of the final MCQs based examinations of all the three courses under consideration for the pre-training (academic years 2012–2013) and post-training (academic years 2013–2014) were calculated. The overall result showed significant improvement of P-value (χ^2^ = 11.61, p = 0.003), however, non-significant improvement of DI values (χ^2^ = 2.27, p = 0.131) was obtained through MCQs for the academic year 2013–2014 and the academic year 2012–2013 for all the three courses (Table 2).

The numbers of non-functional distractors (NFDs) were also less (n = 13) in the year 2013–2014 (post-training) and it was more than double in case of the year 2012–2013 (pre-training) (n = 28) (χ^2^ = 6.0, p = 0.02). The MCQs were further divided into difficult, moderate and easy categories based on their difficulty index (Table 3). The numbers of moderately difficult MCQs were more (n = 183) in the year 2013–2014 (post-training) as compared to the year 2012–2013 (pre-training) (Table 2). The numbers of easy type MCQs were quite less.

On the basis of Bloom's cognitive levels, in the academic year 2013–2014 (post-training) the K2 MCQs were more (n = 123) in comparison to K2 level MCQs (n = 84) in the academic year 2012–2013 (pre-training), whereas K1 MCQs were decreased to 117 from 156 after training (χ^2^ = 12.91, p<0.0001). The MCQs with item writing flaws (IWFs) were around ten times more in numbers (n = 47) before workshop training program and reduced significantly (n = 5) after the training program (χ^2^ = 38.04, p <0.0001). The items analysis showed significant variation between the pre- and post-training in the P-values of respiratory (F = 4.964, p = 0.027), cardiovascular (F = 6.253, p = 0.013) and renal block examinations (F = 7.852, p = 0.006) (Table 3). Similarly, item writing flaws also have significant variation between pre- and post-training in all three block examination (Table 3).

## Discussion

Many untrained tutors are excellent in their academic responsibilities but earlier findings proved that medical faculties can be more effective in their roles with formal training^21^. FDPs built professional development especially for new faculties members to their various academic roles. Therefore, FDPs activities will appear highly valuable and effective, if participant's outcome measured to changes in learning, behavior and performances^22^. Our results show effectiveness of MCQs items' writing workshop training in positive context to items related outcome, student's mean score and passing rate (Table 1and Table 2). The results analysis showed a significant positive difference in the measured outcomes including DI and p-values of the final MCQs based examinations of all the three courses separately included in this analysis for the academic year 2013–2014 (post-training) over the academic year 2012–2013 (pre-training). The difference in pre- and post-training DI values suggests that the faculty development activity in the academics of medicine resulted in significant improvement in the quality of test items development by the participants. Significant differences were found for DIs in pre- and post-training examination, as more MCQs were present in all the three courses and showed significant improvement after faculty development program and demonstrated that the quality of the MCQs were improved after attending the FDP by the participants. Overall improvement of MCQs items' writing skills pre- to post workshop training reflect increased mean score and passing rate of students. Our results were in close agreement with the earlier reports of Naeem et al. and Jozefowicz et al.^18^,^23^. Naeem et al. evaluated the effect of FDPs on quality of MCQs, short answer questions (SAQ) and objective structured clinical examination (OSCE) items writing and reported significant improvement in the quality of test items developed by the participants after training intervention^18^. They achieved high effect sizes, which indicate strong effect of the dedicated FDP on items' quality^18^. Whereas, Jozefowicz et al. reported that the United State Medical Licensing Examination (USMLE) Step-1 questions written by trained faculties had mean score higher as compared to the faculties without formal training^23^. Other studies also demonstrated the benefit of both peer review and structured training in improving item writing quality^24^,^25^. In contrast, based on the utility and modern trend of medical school examinations' item writing our study was only focused on in-house MCQs items development. Also, we evaluated the effect of FDPs on MCQs items' writing quality in terms of increased mean score and passing rate of students. By applying rigorous statistical analysis on training intervention, we found significant decrease in easy questions, NFDs and IWFs, whereas remarkable improvement was noticed in DIs and Bloom's taxonomy, thus making our findings more reliable and significant from analytical point of view.

Our result indicated that the pre- and post-training reliability (Kr-20) of the examination was >0.92, which indicates homogeneity of the test. It was also confirmed that the reliability of the test is not only depends upon the quality of the MCQs but also on the number of MCQs, distribution of the grades and the time provided for examinations^26^.

MCQs with a higher number of NFDs are easier than those with a lower number of NFDs and are less discriminating items^13^,^27^,^28^. Distractors usually distract the less knowledgeable student, but they should not result in tricky questions which might mislead knowledgeable examinees. A question with only two good distractors, however, is preferable to one with additional filler options added only to make up some pre-determined number of options^10^. We also found that the number of NFDs were less in the MCQs of post-training examination. The discriminatory power of a MCQ is largely depends upon the quality of its distractors. An effective distractor will look plausible to less knowledgeable students and lure them away from the keyed option but, it will not entice students who are well-informed about the topic under consideration. Writing effective distractors can be a challenging job, but helpful guidelines that can make the process easier are readily available^29^,^30^.

Assessment derives learning^3^,^31^, which should be perfectly intervened with higher levels of cognitive abilities. The ‘learning approach’ is a dynamic characteristic and is continuously modified according to students' perception of the learning environment^32^. The cognitive level of the assessment should be in line with the cognitive level of the course objectives and with the instructional activities and the materials provided to students, which is known as ‘constructive alignment of assessment’^33^.

One of the most common problems that affect the MCQs' quality is the presence of item writing flaws (IWFs). The IWFs are any type of violations of accepted/standard item-writing guidelines that can affect students' performance on MCQs and making the item either easier or sometimes even more difficult^11^. Various researchers have identified potential reasons for lack of quality questions and they reported IWFs as one of the major reasons. Vyas and Supe (2008)^34^ reported that limited time and lack of faculty training in the area of MCQs preparation significantly contribute to flaws in writing quality items.

Downing (2005)^11^ assessed the quality of four examinations given to medical students in the United States of America and found that 46% of MCQs contained IWFs and reported that as a consequence of these IWFs, 10–15% of students who were classified as failures would have been classified as pass if MCQs' items with IWFs were removed. Earlier, it has been reported that if the flaws items had been removed from the test, fewer lower achieving examinees would have been passed the test and higher achieving examinees would have obtained high scores^35^. These results were consistent with our findings of decreased IWFs and increased passing rate in pre- to post-test training intervention. The flawed items may also affect difficulty and discrimination index. Low difficulty and poor discrimination in an item favors low achievers, whereas high difficulty and poor discrimination negatively affect the high scorers^36^. Moreover, flawed items also fail to assess the courses learning objectives^36^.

**Figure 1:**
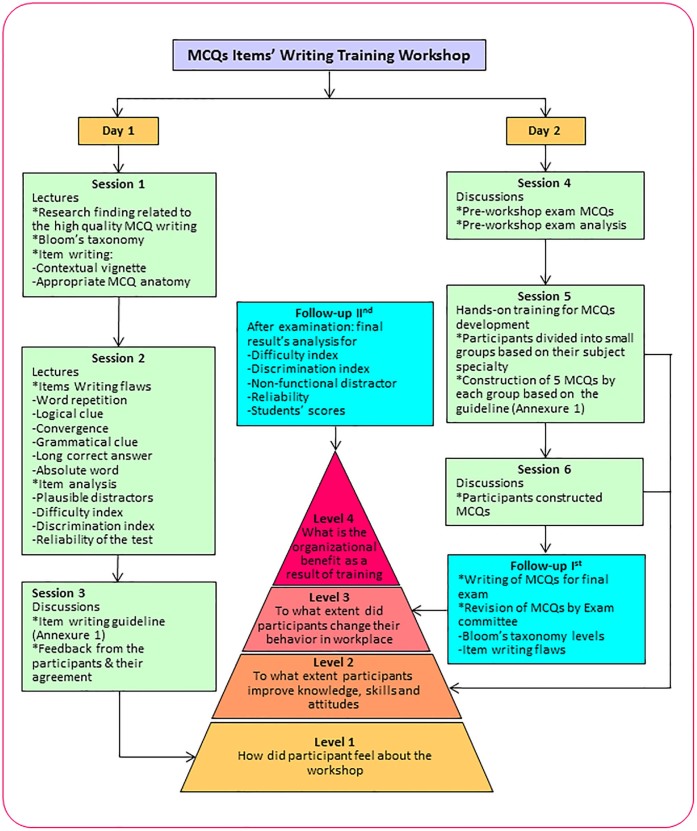
Flow-chart of MCQs items' writing training workshop program structure according to the Kirkpatrick's levels of evaluation (adopted from Abdughani et al., 2014).

The methods of assessment inspire the approach of students towards learning. Students are inclined to expose a surface approach when assessment emphasis is on recall of factual knowledge and students are more likely to adopt a surface approach^37^. The present study concludes that faculty should be encouraged and trained to construct MCQs for higher order cognitive levels to assess trainees in appropriate manner^38^. The current study also paves the way for application of suitable faculty development programs to improve the quality of MCQs for other career options/degrees. Improvement in the quality of MCQs will improve the validity of the examination as well as students' deep learning approaches. The outcomes indicate that FDPs should be arranged regularly in a well-organized format and schedule. A flow-chart of MCQs items' writing training workshop program structure according to the Kirkpatrick's levels of evaluation has been given as a reference (Figure 1).

Several evaluations of FDPs have occurred immediately or soon after the conclusion of the intervention^14^,^16^,^17^,^18^. Few studies have examined the impact of these programs on the skills and behaviors of participants at a later date^39^,^40^,^41^. This is the very first communication reporting the sustainability and the long term effect of FDPs on newly joined faculty members in MCQs construction skills development.

In addition to our progressive findings, certain limitations were associated with this study, such as: (i) the present study only revealed the results of single group of faculties' and students' scores only in three courses of the first year medical degree, (ii) further workshops will be needed for other assessment tools, such as, ‘short answer questions’, ‘objective structured practical examination’ and ‘objective structured clinical examinations’ which are also included in the assessment, (iii) such workshops must be conducted for broader scales on different contexts and variety of examinations.

In conclusion, well-constructed faculty development training improves the quality of the MCQs in terms of difficulty and discriminating indices, Bloom's cognitive levels and reduces item writing flaws and non-functioning distractors. Improvement in quality of MCQs will surely improve the validity of the examination as well as students' better achievements in their assessment. Based upon the outcomes, we can suggest that faculty development trainings should be conducted on regular basis and in a proper manner as well as follow up process for the continuously of the quality assessment process. Such training will lead to greater effectiveness. Also, the effectiveness of training depends more on design (should be aimed for learner's need) and implementation of training program.

## Methodology

### Study context

PNU is the biggest female university of Saudi Arabia and its College of Medicine started functioning from the academic year 2012–2013. Many new faculties joined the College of Medicine, PNU on different academic positions. The Bachelor's medicine degree curriculum of the college is distributed over five years in five phases, i.e., Phase I: preparatory (pre-medical year; one year duration); Phase II: first and second pre-clinical years with normal and abnormal structure and function including subjects of basic sciences (anatomy, histology, embryology, physiology, biochemistry, microbiology, pathology, clinical medicine, sociology and epidemiology, etc.) intertwined with clinical relevance; Phase III & IV: third and fourth clinical years include anesthesia, ENT, dermatology, ophthalmology, orthopedics, primary health care and psychiatry; fifth year deals with medicine, pediatrics and surgery including 4 weeks elective course; and Phase V: consists of rotations and training in the hospital in all required disciplines to complete the internship clinical requirements.

### Participants

A total of 25 faculty members (single group) from the college of medicine, PNU participated in the teaching and assessment of the courses as well as in the study. The MCQs of the final exam were reviewed by the examination committee members of the Assessment and Evaluation Center (AEC), College of Medicine, King Saud University (KSU), Riyadh. A high quality MCQs construction checklist based on pertinent literature review^38^,^42^,^43^,^44^,^45^ was developed by the AEC, College of Medicine, KSU (Appendix 1). The PNU faculties were instructed to follow the MCQs construction checklist in the exam's MCQs items' writing. The examination committee members identified that the faculties did not fulfill uniform standards of the MCQs construction checklist. Therefore, the faculty development unit of PNU introduced two workshops on MCQs items writing which were run by the members of the Faculty Development Unit, Department of Medical Education, College of Medicine, KSU. The workshops contents included interactive didactic sessions along with hands-on MCQs items construction training in the beginning of academic year 2013–2014.

### Faculty development program workshop intervention

Two days full time workshop was conducted for 25 newly joined faculty members of PNU. The workshop was designed to train faculty members, to construct high quality single-best MCQs for basic science courses. The participants were asked to bring five MCQs from their specialties to be discussed in the workshop.

On the first day, theoretical backgrounds were discussed along with the revision of the MCQs construction checklist criteria and consensus was achieved regarding the checklist items with the members. Whenever a disagreement was raised with any checklist item, that was discussed again for its rationale and disagreement was resolved. All pre-workshop MCQs, which were developed, by the faculty members, were revised based on the agreed checklist criteria and corrected accordingly.

On the second day of the workshop the participants were divided into three to four participants, in a small group and asked to develop five MCQs in their specialties, based on the provided and agreed checklist criteria. Further the MCQs were discussed and again, corrected and edited with the participants' agreement.

### Follow–up studies of the workshop intervention

After workshop training intervention, new MCQs were constructed by the faculty members of PNU, for the final examinations of the three courses (respiratory, cardiovascular and renal courses), based on the MCQs construction guideline criteria (Appendix 1). Further MCQs were collected and discussed at the departmental level, College of Medicine (PNU), for final students' assessment. Any MCQ which did not fulfill the agreed guidelines criteria was modified or deleted, accordingly. Our study measured the quality of pre-training (academic year 2012–2013) and post-training (academic year 2013–2014) MCQs construction of above mentioned three courses. A flow-chart of well-structured MCQs items' writing training workshop program has been given as Figure 1.

The main outcomes measures of the study were the results of the final MCQs test items (80 in number in each course) of three courses, i.e., respiratory, cardiovascular and renal for two consecutive academic years 2012–2013 (pre-training) and 2013–2014 (post-training). The outcomes were measured in term of MCQ items construction (Bloom's cognitive levels and presence or absence of the item writing flaws (IWFs)), MCQ items analysis ((Difficulty index (P), Discriminating index (DI), non-functioning distractors (NFDs) and reliability of the tests (Kr-20)), and student's performance (average mean score and overall passing rate).

### Quality measurement of the items of MCQs

The Kirkpatrick's model of educational outcomes offers a useful evaluation framework for the faculty development workshops. The Kirkpatrick's evaluation model has been found very useful in evaluating the workshops with higher level outcomes^13^,^46^. The present study lies in the fourth level of the Kirkpatrick's model which evaluated the change among the participants' performance in the MCQs items' writing outcome at three different levels.

### 1. MCQs items construction in terms of Bloom's cognitive level and Item writing flaws

A well-constructed MCQ consists of a stem (a clinical case scenario), a lead-in (question) and followed by 4–5 choice options (one correct/best answer and three to four distractors^7^,^42^. Bloom's taxonomy divided the cognitive domain into six hierarchically ordered categories: knowledge, comprehension, application, analysis, synthesis, and evaluation^47^. Tarrant et al.^38^ simplified the taxonomy by creating two different levels, i.e., K1 which represents basic knowledge and comprehension; K2 which encompasses application and analysis. MCQ items at K2 level are better, more valid and discriminating good students from poor performing students^13^.

MCQs with IWFs are those items which violate the standard item-writing guidelines. The flawed MCQs test items reduces the validity of examinations and penalizing some examinees^11^. In order to investigate the effectiveness of the faculty development program a checklist was used for checking the quality of MCQs (Appendix 1).

### 2. MCQs item analysis (Difficulty index, Discrimination index, Non-functional distractors and Kr-20)

Difficulty index also named as P-value describes the percentage of students who correctly answered a given test item. This index ranges from 0 to 100% or 0 to 1. An easy item has a higher difficulty index. The cut-off values maintained to evaluate the difficulty index of MCQs were: >70% (easy); 20–70% (moderate); <20% (difficult)^48^. Moderate difficulty items (20–70%) in a test have better discriminating ability^13^.

Discriminating index is the ability of a test item to discriminate between high and low examinee scorers. Higher discriminating indices in a test indicate better and greater discriminating capability of that test. The cut-off values for the discrimination index (DI) were taken as, discriminating index > 0.15, and non-discriminating index ≤ 0.15^27^.

Nonfunctioning distractor (NFD) is an option(s) of a question(s), that have been selected by less than 5% of the examinees^49^. These NFDs may have no connection or have some clues which are not directly related to the correct answer^38^. Implausible distractors can be easily spotted even by the weakest examinees and are therefore usually rejected outright. Distractors that are not chosen or are consistently chosen by only a few participants are obviously ineffective and should be omitted or replaced^50^,^51^.

Kuder-Richardson Formula 20 (Kr-20) measures the internal consistency and reliability of an examination. The KR20 formula is a measure of internal consistency for examinations with dichotomous choices. If Kr-20 coefficient is high (e.g., >0.90), it is an indication of a homogeneous test. If Kr-20 figure is 0.8, it is considered as the minimal acceptable value, whereas figure below 0.8 indicates non-reliability of the exam^52^. Question mark perception software program (Questionmark Corporation, Norwalk, CT, USA) was used for the items' analysis and for the determination of Kr-20.

### 3. Students' performance

The MCQs items writing flaw and plausible distractor affect students' performance. Some flaws, such as the use of unfocused or unclear stems, gratuitous or unnecessary information and negatively worded in the stem can make questions more difficult^35^. Similarly, plausible distractor creates misconception about the correct option, at least in the average examinee's mind^28^.

### Statistical analysis

The data obtained were entered in the Microsoft Excel file and analyzed using SPSS software (version 19.0). Pearson's chi-square test was used to evaluate and quantify the correlation, whereas ANOVA test was used for variance analysis between the categorical outcomes. The statistical significance level was set as p-value < 0.05 during the entire analysis.

### Ethical considerations

The participants were informed about the study and agreed to get involved in the project. The study was approved by the research ethical committees of the respective medical colleges of KSU and PNU, Riyadh, Saudi Arabia. Also, the employed methods for this study were carried out in accordance with the approved guidelines of the respective medical colleges of KSU and PNU, Riyadh, Saudi Arabia.

## Author Contributions

Conceived and designed the study and experiments: H.M.A., F.A., M.I., M.S.K., G.K.A., S.S., A.A.A., A.N. and S.H., Performed the experiments: H.M.A., F.A., M.I. and S.H., Analyzed the data: F.A. and M.I., Contributed reagents/materials/analysis tools: M.S.K., G.K.A., S.S., A.A.A. and A.N., Wrote the paper: H.M.A., F.A., M.I. and S.H.

## Supplementary Material

Supplementary Information Appendix 1

## Figures and Tables

**Table 1:** Specification of examination (Total number of MCQs = 80; Marks = 30)

Courses	Students passed, n(%)	Students failed, n(%)	Mean score	Reliability coefficient
***Pre-training***				
Respiratory	24/64(37.5)	40/64(62.5)	16.89	0.93
Cardiovascular	27/63(42.9)	36/63(57.1)	17.02	0.93
Renal	41/61(67.2)	20/61(32.8)	20.96	0.94
***Post-training***				
Respiratory	46/80(57.5)	34/80(42.5)	18.7	0.92
Cardiovascular	40/73(54.8)	33/73(45.2)	20.94	0.93
Renal	39/69(56.5)	30/69(43.6)	21.31	0.92

**Table 2:** Different factors associated with the item analysis

		Respiratory		Cardiovascular		Renal		All three courses		
		Pre*	Post**	Pre*	Post**	Pre*	Post**	Pre	Post	
Factors	Categories	n(%)	n(%)	n(%)	n(%)	n(%)	n(%)	n(%)	n(%)	χ^2^(p)
Difficulty Index (P)	Difficult (<20%)	1(1.3)	0(0)	0(0)	2(2.5)	1(1.3)	1(1.3)	2(0.8)	3(1.3)	11.61(0.003)
	Moderated (20–70%)	55(68.8)	61(76.3)	59(73.8)	68(85.0)	36(45.0)	54(67.5)	150(62.5)	183(76.3)	
	Easy (>70%)	24(30.0)	19(23.8)	21(26.3)	10(12.5)	43(53.8)	25(31.3)	88(36.7)	54(22.5)	
	Total	80(100)	80(100)	80(100)	80(100)	80(100)	80(100)	240(100)	240(100)	
Discrimination Index (DI)	Non-DI(≤0.15)	6(7.5)	3(3.80)	7(8.8)	4(5.0)	6(7.5)	4(5.0)	19(7.9)	11(4.6)	2.27 (0.13)
	DI(>0.15)	74(92.5)	77(96.3)	73(91.3)	76(95.0)	74(92.5)	76(95.0)	221(92.1)	229(95.4)	
	Total	80(100)	80(100)	80(100)	80(100)	80(100)	80(100)	240(100)	240(100)	
Non-function distractors (NFD)	NFD	4(5.0)	2(2.5)	8(10.0)	7(8.8)	16(20.0)	4(5.0)	28(11.7)	13(5.4)	6.00 (0.02)
	FD***	76(95.0)	78(97.5)	72(90.0)	73(91.3)	64(80.0)	76(95.0)	212(88.3)	227(94.6)	
	Total	80(100)	80(100)	80(100)	80(100)	80(100)	80(100)	240(100)	240(100)	
Bloom's taxonomy levels	K1	45(56.3)	40(50.0)	54(67.5)	40(50.0)	57(71.3)	37(46.3)	156(65.0)	117(48.8)	12.91 (<0.0001)
	K2	35(43.8)	40(50.0)	26(32.5)	40(50.0)	23(28.8)	43(53.8)	84(35.0)	123(51.3)	
	Total	80(100)	80(100)	80(100)	80(100)	80(100)	80(100)	240(100)	240(100)	
Items writing flaws (IWFs)	IWF	22(27.5)	1(1.3)	15(18.8)	0(0.0)	10(12.5)	4(5.0)	47(19.6)	5(2.1)	38.04 (0.<0001)
	Without-IWF	58(72.5)	79(98.8)	65(81.3)	80(100)	70(87.5)	76(95.0)	193(80.4)	235(97.9)	
	Total	80(100)	80(100)	80(100)	80(100)	80(100)	80(100)	240(100)	240(100)	

*Pre-training, **Post-training, ***Functional distractor, K1 = Non-scenario bases, K2 = Scenario bases.

**Table 3:** Analysis of variance between pre- and post-training test items

Subjects	Factors	F	ANOVA (p)
Respiratory	Difficulty Index (P)	4.964	0.027
	Discrimination Index (DI)	1.053	0.306
	Non-function distractors (NFD)	0.687	0.408
	Bloom's taxonomy levels	0.622	0.431
	Items writing flaws (IWF)	25.711	0.000
Cardiovascular	Difficulty Index (P)	6.253	0.013
	Discrimination Index (DI)	0.872	0.352
	Non-function distractors (NFD)	0.073	0.788
	Bloom's taxonomy levels	5.154	0.025
	Items writing flaws (IWF)	18.231	0.000
Renal	Difficulty Index (P)	7.852	0.006
	Discrimination Index (DI)	0.422	0.517
	Non-function distractors (NFD)	8.566	0.004
	Bloom's taxonomy levels	10.889	0.001
	Items writing flaws (IWF)	3.833	0.050
